# *K*-Optimal Gradient Encoding Scheme for Fourth-Order Tensor-Based Diffusion Profile Imaging

**DOI:** 10.1155/2015/760230

**Published:** 2015-09-14

**Authors:** Mohammad Alipoor, Irene Yu-Hua Gu, Andrew Mehnert, Stephan E. Maier, Göran Starck

**Affiliations:** ^1^Department of Signals and Systems, Chalmers University of Technology, 41296 Gothenburg, Sweden; ^2^Centre for Microscopy, Characterisation and Analysis, The University of Western Australia, Perth, WA 6009, Australia; ^3^Department of Radiology, Sahlgrenska University Hospital, Gothenburg University, 41345 Gothenburg, Sweden; ^4^Department of Radiation Physics, Institute of Clinical Sciences, University of Gothenburg, 41345 Gothenburg, Sweden; ^5^Department of Medical Physics and Biomedical Engineering, Sahlgrenska University Hospital, 41345 Gothenburg, Sweden

## Abstract

The design of an optimal gradient encoding scheme (GES) is a fundamental problem in diffusion MRI. It is well studied for the case of second-order tensor imaging (Gaussian diffusion). However, it has not been investigated for the wide range of non-Gaussian diffusion models. The optimal GES is the one that minimizes the variance of the estimated parameters. Such a GES can be realized by minimizing the condition number of the design matrix (*K*-optimal design). In this paper, we propose a new approach to solve the *K*-optimal GES design problem for fourth-order tensor-based diffusion profile imaging. The problem is a nonconvex experiment design problem. Using convex relaxation, we reformulate it as a tractable semidefinite programming problem. Solving this problem leads to several theoretical properties of *K*-optimal design: (i) the odd moments of the *K*-optimal design must be zero; (ii) the even moments of the *K*-optimal design are proportional to the total number of measurements; (iii) the *K*-optimal design is not unique, in general; and (iv) the proposed method can be used to compute the *K*-optimal design for an arbitrary number of measurements. Our Monte Carlo simulations support the theoretical results and show that, in comparison with existing designs, the *K*-optimal design leads to the minimum signal deviation.

## 1. Introduction

Diffusion-weighted MRI is a noninvasive imaging technique to probe microstructures in living tissues, for example, the human brain. It involves acquiring a series of diffusion-weighted images, each corresponding to diffusion sensitization along a particular gradient direction. Non-Gaussian diffusion models have gained wide attention among researchers because of their potential ability to resolve complex multifiber microstructures. Özarslan and Mareci [1] introduced high order tensors (HOTs) as an alternative to conventional second-order tensor model. In regions with complex microstructures, HOTs can model the apparent diffusion coefficient (ADC) with higher accuracy than the conventional second-order model [2]. Several aspects of HOT-based ADC profile estimation have been addressed in the literature [3–5]. HOTs have also been used to represent orientation distribution functions that are required for tractography [6, 7].

The need for robust estimation of diffusion parameters in a limited acquisition time has given rise to many studies on optimal gradient encoding scheme (GES) design. In the case of the classical second-order model they include [8–14]. However, there are few studies tackling the problem of optimal GES design for non-Gaussian diffusion models [15, 16]. The only study on GES design for HOTs [16] is limited to comparison of existing GESs mainly devised for second-order tensor imaging, for example, the minimum condition number (MCN) scheme [12]. A caveat here is that the condition number is computed from the design matrix associated with the linear least square estimation of parameters of interest. Thus, by definition, it is model-dependent. This implies that the minimum condition number GES for second-order tensor estimation is not an optimal GES for fourth-order tensor estimation. An experiment design that minimizes the condition number of the design matrix is called *K*-optimal design. In this paper we solve the problem of *K*-optimal GES design for HOT-based ADC profile imaging as follows. First, we reformulate it as a nonconvex experiment design problem. Then, by convex relaxation we obtain a tractable semidefinite programming (SDP) problem. The last step is to extract design points (the gradient encoding directions) from the optimal design matrix. Finally, to show the relevance of the proposed design approach, we evaluate our solutions using the rotational variance test and Monte Carlo simulations. Throughout the paper “*experiment design*” and “*gradient encoding scheme (GES)*” are used interchangeably. The former is used in the optimization context while the latter is used in the diffusion MRI (dMRI) community.

## 2. Problem Statement

This section briefly reviews the basics of HOT-based ADC profile estimation both for the sake of completeness and to define notation. The reader is referred to [4, 5] for more details. For definitions of symmetry, positive semidefiniteness and eigendecomposition of high order tensors (*m* ≥ 4) see [5, 17]. The Stejskal-Tanner equation for dMRI signal attenuation is [18]

(1) $$\begin{array}{c} -\frac{1}{b}\ln\left(\;\frac{S\left(g_{i}\right)}{S_{0}}\;\right)=d\left(\;g_{i}\;\right), \end{array}$$

where *d*(**g**_*i*_) is the diffusivity function, *S*(**g**_*i*_) is the measured signal when the diffusion sensitizing gradient is applied in the direction $g_{i}=\left[\begin{array}{ccc} x_{i} & y_{i} & z_{i} \end{array}\right]$, *S*_0_ is the observed signal in the absence of such a gradient, and *b* is the diffusion weighting taken to be constant over all measurements. The diffusivity function *d*(**g**_*i*_) is modeled using even order symmetric positive semidefinite tensors as follows:

(2) $$\begin{array}{c} d\left(\;g_{i}\;\right)=t^{T}a_{i}, \end{array}$$

where **t** ∈ *ℝ* ^(*n*+1)(*n*+2)/2^ contains distinct entries of the *n*th-order tensor. Here we focus on the case of *n* = 4, where **a**_*i*_ = [*z*_*i*_^4^ 4*y*_*i*_ *z*_*i*_^3^ 6*y*_*i*_^2^ *z*_*i*_^2^ 4*y*_*i*_^3^ *z*_*i*_ *y*_*i*_^4^ 4*x*_*i*_ *z*_*i*_^3^ 12*x*_*i*_ *y*_*i*_ *z*_*i*_^2^ 12*x*_*i*_ *y*_*i*_^2^ *z*_*i*_ 4*x*_*i*_ *y*_*i*_^3^ 6*x*_*i*_^2^ *z*_*i*_^2^ 12*x*_*i*_^2^ *y*_*i*_ *z*_*i*_ 6*x*_*i*_^2^ *y*_*i*_^2^ 4*x*_*i*_^3^ *z*_*i*_ 4*x*_*i*_^3^ *y*_*i*_ *x*_*i*_^4^]^*T*^. It is worth mentioning that both vectors **t** and **a**_*i*_ are vectors in *ℝ* ^15^ and *d*(**g**_*i*_, **t**) = *d*(**g**_*i*_) is used for simplification. Given measurements in *N* > 15 different directions **g**_*i*_, the least squares estimator (LSE) of the HOT is obtained as follows:

(3) $$\begin{array}{c} \underset{t\in R^{15}}{\min}{\left(\;s-Gt\;\right)}^{T}\left(\;s-Gt\;\right), \end{array}$$

where **G** is an *N* × 15* design matrix* defined as $G={\left[\begin{array}{cccc} a_{1} & a_{2} & \cdots & a_{N} \end{array}\right]}^{T}$ and *s*_*i*_ = −*b* ^−1^ln⁡(*S*(**g**_*i*_)/*S*_0_). The closed-form solution is $\hat{t}={\left(G^{T}G\right)}^{-1}G^{T}s$.

In the framework described above, the precision of the estimation problem is dependent on the experiment designs **a**_*i*_, *i* = 1,…, *N*. For independent and zero mean measurement noise with constant variance *σ* ^2^ the LSE is unbiased and has the following covariance matrix [19]:

(4) $$\begin{array}{c} \mathrm{cov}\left(\;\hat{t}\;\right)=\sigma^{2}M^{-1}, \end{array}$$

where **M** = **G** ^*T*^ **G** = ∑_*i*=1_^*N*^ **a**_*i*_ **a**_*i*_^*T*^ and is usually called the “*information matrix*.” Optimal experiment design entails making the covariance matrix* small* in some sense. It is usual to minimize a scalar function of the covariance matrix. One design approach is to minimize the condition number of the information matrix (*K*-optimal design) [11, 12, 19]. In this paper, we solve the *K*-optimal experiment design problem for HOT-based ADC profile imaging.

**Remark 1 .**

For isotropic diffusion, it has been shown that (4) holds [9, 20]. We investigate the significance of the noise assumptions in the case of anisotropic diffusion, later in Section 4. Therein we present Monte Carlo simulations for a more realistic case (with anisotropic tensor and Rician distributed noise on *S*(**g**_*i*_)).

## 3. Proposed GES Design Approach

In Section 3.1 we present mathematical formulations for the *K*-optimal GES design problem. The solutions are given in Section 3.2.  Section 3.3 then considers the problem of extracting the design points from the optimal information matrix. Finally, the properties of the obtained solutions and some theoretical results are discussed in the last subsection.

### 3.1. Mathematical Formulations of the *K*-Optimal Design Problem

The condition number measures the sensitivity of the solution to changes in measurements [11]. Hence, it is desirable to minimize the condition number of **M** ^−1^ (denoted by *κ*(**M** ^−1^)) or equivalently to minimize *κ*(**M**). The *K*-optimal design in HOT-based ADC profile imaging can be performed with respect to either the design matrix **G** or information matrix **M** because [11]

(5) $$\begin{array}{c} \kappa \left(\;G\;\right)=\sqrt{\kappa \left(M\right)}=\sqrt{\frac{\lambda_{\max}\left(M\right)}{\lambda_{\min}\left(M\right)}}, \end{array}$$

where *λ*_max_(**M**) and *λ*_min_(**M**) are the maximum and minimum eigenvalues of **M**, respectively. The *K*-optimal experiment design problem in HOT estimation can be written as follows:

(6) min⁡gi κMs.t.: M≥0, gi=1,i=1,…,N.

This problem, in its current form, is not convex. Our aim here is to reformulate this problem as an SDP problem that can be efficiently solved. Before describing the approach, it is worth mentioning that conventional experiment design problems (as in [21]) seek to minimize the objective function over a finite and thus countable set *𝒜*, that is, ∀*i* : **a**_*i*_ ∈ *𝒜*. In the present case, however, *𝒜* is not a countable set but includes the whole set of feasible solutions. Note that the degree of freedom in this design problem is 45. In other words, **M** can be parameterized in 45 independent variables. For example, *m*_1,2_ = *m*_2,1_, *m*_10,10_ = 36*m*_1,2_, *m*_4,6_ = *m*_6,4_ = 16*m*_1,2_. To reformulate the problem, we first parameterize **M** in 45 distinct variables as

(7) $$\begin{array}{c} M=\left[\begin{array}{ccccccccccccccc} q_{1} & q_{10} & q_{12} & 4q_{16} & 4q_{17} & 4q_{18} & 4q_{19} & 4q_{20} & 4q_{21} & 6q_{6} & 6q_{15} & 6q_{7} & 12q_{22} & 12q_{23} & 12q_{24}\\ q_{10} & q_{2} & q_{11} & 4q_{25} & 4q_{26} & 4q_{27} & 4q_{28} & 4q_{29} & 4q_{30} & 6q_{4} & 6q_{9} & 6q_{14} & 12q_{31} & 12q_{32} & 12q_{33}\\ q_{12} & q_{11} & q_{3} & 4q_{34} & 4q_{35} & 4q_{36} & 4q_{37} & 4q_{38} & 4q_{39} & 6q_{13} & 6q_{8} & 6q_{5} & 12q_{40} & 12q_{41} & 12q_{42}\\ 4q_{16} & 4q_{25} & 4q_{34} & 16q_{4} & 16q_{43} & 16q_{10} & 16q_{21} & 16q_{26} & 16q_{32} & 24q_{27} & 24q_{31} & 24q_{44} & 48q_{14} & 48q_{33} & 48q_{28}\\ 4q_{17} & 4q_{26} & 4q_{35} & 16q_{43} & 16q_{5} & 16q_{20} & 16q_{12} & 16q_{40} & 16q_{34} & 24q_{45} & 24q_{41} & 24q_{37} & 48q_{42} & 48q_{13} & 48q_{36}\\ 4q_{18} & 4q_{27} & 4q_{36} & 16q_{10} & 16q_{20} & 16q_{6} & 16q_{24} & 16q_{45} & 16q_{28} & 24q_{16} & 24q_{44} & 24q_{22} & 48q_{15} & 48q_{21} & 48q_{23}\\ 4q_{19} & 4q_{28} & 4q_{37} & 16q_{21} & 16q_{12} & 16q_{24} & 16q_{7} & 16q_{36} & 16q_{44} & 24q_{23} & 24q_{45} & 24q_{17} & 48q_{20} & 48q_{15} & 48q_{22}\\ 4q_{20} & 4q_{29} & 4q_{38} & 16q_{26} & 16q_{40} & 16q_{45} & 16q_{36} & 16q_{8} & 16q_{11} & 24q_{43} & 24q_{39} & 24q_{42} & 48q_{41} & 48q_{34} & 48q_{13}\\ 4q_{21} & 4q_{30} & 4q_{39} & 16q_{32} & 16q_{34} & 16q_{28} & 16q_{44} & 16q_{11} & 16q_{9} & 24q_{33} & 24q_{29} & 24q_{43} & 48q_{26} & 48q_{31} & 48q_{14}\\ 6q_{6} & 6q_{4} & 6q_{13} & 24q_{27} & 24q_{45} & 24q_{16} & 24q_{23} & 24q_{43} & 24q_{33} & 36q_{10} & 36q_{14} & 36q_{15} & 72q_{44} & 72q_{28} & 72q_{21}\\ 6q_{15} & 6q_{9} & 6q_{8} & 24q_{31} & 24q_{41} & 24q_{44} & 24q_{45} & 24q_{39} & 24q_{29} & 36q_{14} & 36q_{11} & 36q_{13} & 72q_{34} & 72q_{26} & 72q_{43}\\ 6q_{7} & 6q_{14} & 6q_{5} & 24q_{44} & 24q_{37} & 24q_{22} & 24q_{17} & 24q_{42} & 24q_{43} & 36q_{15} & 36q_{13} & 36q_{12} & 72q_{36} & 72q_{45} & 72q_{20}\\ 12q_{22} & 12q_{31} & 12q_{40} & 48q_{14} & 48q_{42} & 48q_{15} & 48q_{20} & 48q_{41} & 48q_{26} & 72q_{44} & 72q_{34} & 72q_{36} & 144q_{13} & 144q_{43} & 144q_{45}\\ 12q_{23} & 12q_{32} & 12q_{41} & 48q_{33} & 48q_{13} & 48q_{21} & 48q_{15} & 48q_{34} & 48q_{31} & 72q_{28} & 72q_{26} & 72q_{45} & 144q_{43} & 144q_{14} & 144q_{44}\\ 12q_{24} & 12q_{33} & 12q_{42} & 48q_{28} & 48q_{36} & 48q_{23} & 48q_{22} & 48q_{13} & 48q_{14} & 72q_{21} & 72q_{43} & 72q_{20} & 144q_{45} & 144q_{44} & 144q_{15} \end{array}\right] \end{array}$$

such that we obtain the affine mapping **M** : *ℝ* ^45^ → *𝕊*_+_^15^ (its range is the set of symmetric positive semidefinite matrices of size fifteen). This can equivalently be expressed as

(8) $$\begin{array}{c} M\left(\;q\;\right)=q_{1}M_{1}+\cdots +q_{45}M_{45}, \end{array}$$

where **M**_*j*_ is a 15 × 15 symmetric matrix and **q** ∈ *ℝ* ^45^. To clarify how **M** is decomposed into **M**_*j*_s, consider the following example:

(9) $$\begin{array}{c} {\left[\;M_{10}\;\right]}_{kl}=\left\{\begin{array}{cc} 1 & k=1,\;\;l=2\\ 1 & k=2,\;\;l=1\\ 16 & k=4,\;\;l=6\\ 16 & k=6,\;\;l=4\\ 36 & k=10,\;\;l=10\\ 0 & \text{otherwise}, \end{array}\right. \end{array}$$

where [**M**_10_]_*kl*_ is the element of **M**_10_ placed in the *k*th row and *l*th column. Carefully note the relationship between **q** and the original design variables (**g**_*i*_s) because this is used in Section 3.3. For example, *q*_1_ = ∑*x*_*i*_^8^ and *q*_10_ = ∑*x*_*i*_^4^ *y*_*i*_^4^.

It is possible to relax the constraints ‖**g**_*i*_‖ = 1, *i* = 1,…, *N*, and solve the problem by the algorithm given in [22] to obtain a lower bound on the optimal value of problem (6). However, we instead convert the constraints in (6) to a convex constraint as follows:

(10) gi1,i=1,…,N∑i=1NgiTgi4=N,∑j=13qj+4∑j=49qj+6∑j=1012qj+12∑j=1315qj=NuTq=N,

where **u** ∈ *ℝ* ^45^ has only fifteen nonzero elements. We then have the following relaxed problem:

(11) min⁡q κMqs.t.: Mq≥0, uTq=N.

Given that the conversion in (10) is not reversible, the optimal value of the problem in (11) is a lower bound on the optimal value of the problem in (6). The objective function *κ*(·) is a quasiconvex function [22]. Thus, an approximate solution of (11) may be obtained by solving a sequence of feasibility problems [21, 22]. Alternatively, this problem can be formulated as an SDP problem:

(12) min⁡q,p αs.t.: Mq≥0, I≤pMq≤αIp≥0, uTq=N,

where **I** is the identity matrix, *α* is the condition number, and *p* equals 1/*λ*_min_(**M**). This is a bilinear matrix inequality problem that can be solved by the line search method. For a constant *p*, it becomes a tractable linear matrix inequality (LMI) problem. The optimal value *α* ^*∗*^ of (12) can be obtained by performing a line search on *p*. Let the optimal value of the following problem be *α*_*c*_^*∗*^, where *c* is a real nonnegative constant:

(13) min⁡q αs.t.: Mq≥0, I≤cMq≤αI, uTq=N.

Then we have *α* ^*∗*^ = min{*α*_*c*_^*∗*^∣*c* ∈ *ℝ*_+_}. The problem in (13) can be efficiently solved by LMI solvers.

### 3.2. Solutions to the *K*-Optimal Design Problem

The *K*-optimal design problem in (13) can be solved for different values of *c* ≥ 0, *N* ≥ 15 using the YALMIP [23] and SDPT3 solvers [24]. By close inspection of the results for different values of *N*, one can conclude the following about *K*-optimal solutions:

(i) If **q**_*N*_^*∗*^ is a solution to (13) with **u** ^*T*^ **q**_*N*_^*∗*^ = *N*, then *w* **q**_*N*_^*∗*^ is a solution to (13) with *w* **u** ^*T*^ **q**_*N*_^*∗*^ = *wN* for any real positive *w*. Thus, the optimal solution (**q**_*N*_^*∗*^) is proportional to *N*.
(ii) The minimum condition number is independent of *N* and is given by *α* ^*∗*^ = 3.6639.
(iii) The *K*-optimal solution is
  (14) $$\begin{array}{c} q_{j}^{*}=\left\{\begin{array}{cc} \frac{N}{4.5248} & j=1,2,3\\ \frac{N}{101.8849} & 4\leq j\leq 9\\ \frac{N}{248.2622} & j=10,11,12\\ \frac{N}{1245.3300} & j=13,14,15\\ 0 & 16\leq j\leq 45. \end{array}\right. \end{array}$$
(iv) And $\kappa (G_{K}^{*})=\sqrt{\kappa (M_{K}^{*})}=1.9141$ (where **M**_*K*_^*∗*^ = **M**(**q** ^*∗*^)).

### 3.3. Extracting Design Points

The task of extracting the design points ($\left[\begin{array}{ccc} x_{i} & y_{i} & z_{i} \end{array}\right]$, *i* = 1,…, *N*) from the optimal information matrix **M**(**q** ^*∗*^) is straight forward, as outlined in [25]. By expressing the optimal **M**(**q** ^*∗*^) in terms of the original decision variables, one obtains 45 equations as listed in (15) and (16). Furthermore, *N* equations of the form *x*_*i*_^2^ + *y*_*i*_^2^ + *z*_*i*_^2^ = 1 can be added to guarantee that the resulting solutions belong to the feasible set of the original problem. Thus, one obtains a nonlinear system of 45 + *N* equations in 3*N* unknowns. Given that *N* ≥ 15 is required, the system is usually underdetermined. By numerically solving the nonlinear system, one can extract the design points. The odd moments of the optimal design must be zero (**M** = **M**(**q** ^*∗*^)). We refer to this fact as* symmetry* of the optimal design. This property means that the following holds true:

(15) ∑xi3yi5∑yi3xi5=∑zi3yi5=∑yi3zi5=∑xi3zi5=∑zi3xi5=0,∑xi7yi∑yi7xi=∑zi7yi=∑yi7zi=∑xi7zi=∑zi7xi=0,∑yi2xi5zi∑yi2xizi5=∑zi2xi5yi=∑zi2xiyi5=∑xi2yi5zi=∑xi2yizi5=0,∑yi3xi4zi∑yi4xi3zi=∑zi3xi4yi=∑zi4xi3yi=∑xi3yi4zi=∑xi4yi3zi=0,∑yi2xi3zi3∑zi2xi3yi3=∑xi2yi3zi3=0,∑yi6xizi∑zi6xiyi=∑xi6yizi=0.

The even moments of the optimal design must satisfy the following conditions (**M** = **M**(**q** ^*∗*^)):

(16) ∑xi8∑yi8=∑zi8=N4.5248,∑xi4yi4∑xi4zi4=∑zi4yi4=N248.2622,∑yi4xi2zi2∑zi4xi2yi2=∑xi4yi2zi2=N1245.3300,∑xi2yi6∑xi6yi2=∑xi2zi6=∑xi6zi2=∑zi2yi6=∑zi6yi2=N101.8849.

As an example, Table 1 lists the *K*-optimal design points for *N* = 30 derived using our proposed method. We solved the above-mentioned nonlinear system of equations using the fsolve command in MATLAB. For a discussion on the uniqueness of this solution, see the next subsection where we explain some properties of the *K*-optimal design.

### 3.4. Properties and Theoretical Results

*(1) Global Optimality*. In summary, our approach to find the *K*-optimal experiment design involves the following steps. We begin with the original formulation of the *K*-optimal experiment design problem as in (6). Next we apply the relaxation in (10). Finally we solve the relaxed version of the problem as stated in (13). Any point in the feasible set of the original minimization problem gives an upper bound (UB) on the optimal value of its objective function. The optimal point in the feasible set of the relaxed problem gives a lower bound (LB) on the optimal value of the original objective function. Thus, if an optimal solution of the relaxed problem belongs to the feasible set of the original problem, which implies that UB = LB, then it is a globally optimal solution of the original problem. By construction, this is the case for the proposed solutions in previous subsection.

*(2) Relation with Number of Measurements.* As mentioned above, elements of the optimal information matrix are proportional to the number of available measurements *N*. However, the optimal value of the objective function (condition number) is constant.

*(3) Symmetry.* The presented *K*-optimal design is symmetric in the sense specified in (15).

*(4) Nonuniqueness.* The *K*-optimal design is not unique. Let **M**_*N*_*m*__^*∗*^ be the *K*-optimal information matrix when a total of *N*_*m*_ measurements is permitted. Let *ζ*_*N*_*m*__ = {**g**_*i*_∣‖**g**_*i*_‖ = 1, *i* = 1,…, *N*_*m*_} be the set of corresponding design points. If *N*_3_ = *N*_1_ + *N*_2_, then one can easily verify that *ζ*_*N*_1__ ∪ *ζ*_*N*_2__ will result in the same information matrix as *ζ*_*N*_3__. This is because of the linear dependency of the elements of the optimal **M** on *N*, so that **M**_*N*_3__^*∗*^ = **M**_*N*_1__^*∗*^ + **M**_*N*_2__^*∗*^. As an example, for *N*_3_ = 60 one can find the following optimal designs: *ζ*_40_ ∪ *ζ*_20_, *ζ*_15_ ∪ *ζ*_45_, *ζ*_30_ ∪ *ζ*_30_, and even four repetitions of *ζ*_15_.

*(5) Consistency with Previous Studies.* The *K*-optimal design problem for second-order DTI has been studied in [12]. Therein, the solution is approximated using the downhill simplex method (a stochastic optimization method). Using the proposed approach for second-order DTI (set **a**_*i*_ = [*x*_*i*_^2^ *y*_*i*_^2^ *z*_*i*_^2^ 2*x*_*i*_ *y*_*i*_ 2*x*_*i*_ *z*_*i*_ 2*y*_*i*_ *z*_*i*_]^*T*^, **M** = ∑_*i*=1_^*N*^ **a**_*i*_ **a**_*i*_^*T*^, and repeat the whole process in Section 3) one can see that, for an arbitrary *N*, the optimal condition number of the design matrix is $\sqrt{7/4}=1.3229$. The *K*-optimal GES for *N* = 6 is listed in Table 2. All these findings are in agreement with the results in [12].

**Remark 2 .**

The set of second-order tensors can be seen as a subset of fourth-order tensors. As an example, the equality **g** ^*T*^ **D** **g** = **g** ^*T*^ **D** **g**(**g** ^*T*^ **g**) implies that the second-order tensor **D** can be represented by a fourth-order tensor **t** = [*d*_33_ 0.5*d*_23_ (*d*_33_ + *d*_22_)/6 0.5*d*_23_ *d*_22_ 0.5*d*_13_ *d*_12_/6 *d*_13_/6 0.5*d*_12_ (*d*_33_ + *d*_11_)/6 *d*_23_/6 (*d*_11_ + *d*_22_)/

6 0.5*d*_13_ 0.5*d*_12_ *d*_11_], where *d*_*ij*_ denotes the elements of **D**. In such cases, the number of free parameters of the fourth-order tensor is reduced to six, and thus it can be estimated using the *K*-optimal designs for second-order DTI. See Table 2 for an example with *N* = 6.

## 4. Evaluations and Results

In this section we evaluate the proposed *K*-optimal GES in comparison to several existing methods. The evaluation framework is adopted from [16]. More specifically we consider two quality measures: condition number and signal deviation.

### 4.1. Condition Number

Table 3 shows that the proposed *K*-optimal GES has the minimum condition number. References for the competing GESs are also provided in this table.

### 4.2. Signal Deviation

**Figure 1:**
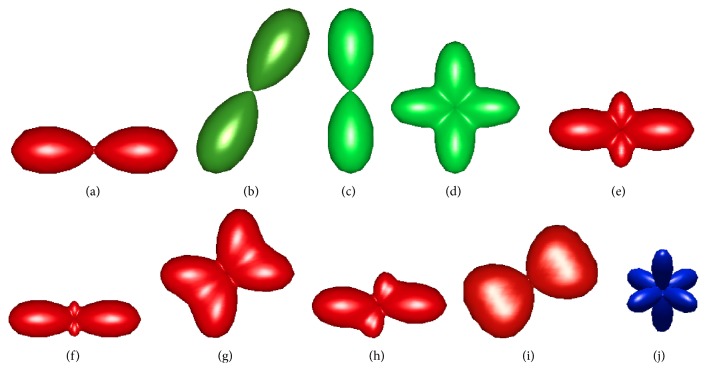
Shape of 10 fourth-order tensors used for the evaluation of the proposed method: (a) single-fiber with orientation $\left[\begin{array}{ccc} 1 & 0 & 0 \end{array}\right]$, (b) single-fiber with orientation $\left[\begin{array}{ccc} \cos60^{{}^{\circ}} & \sin60^{{}^{\circ}} & 0 \end{array}\right]$, (c) single-fiber with orientation $\left[\begin{array}{ccc} 0 & 1 & 0 \end{array}\right]$, (d) two fibers with orientations $\left[\begin{array}{ccc} 1 & 0 & 0 \end{array}\right]$ and $\left[\begin{array}{ccc} 0 & 1 & 0 \end{array}\right]$ and with relative weights 1 : 1, (e) two fibers with orientations $\left[\begin{array}{ccc} 1 & 0 & 0 \end{array}\right]$ and $\left[\begin{array}{ccc} 0 & 1 & 0 \end{array}\right]$ and with relative weights 2 : 1, (f) two fibers with orientations $\left[\begin{array}{ccc} 1 & 0 & 0 \end{array}\right]$ and $\left[\begin{array}{ccc} 0 & 1 & 0 \end{array}\right]$ and with relative weights 4 : 1, (g) two fibers with orientations $\left[\begin{array}{ccc} 1 & 0 & 0 \end{array}\right]$ and $\left[\begin{array}{ccc} \cos75^{{}^{\circ}} & \sin75^{{}^{\circ}} & 0 \end{array}\right]$ and with relative weights 1 : 1, (h) two fibers with orientations $\left[\begin{array}{ccc} 1 & 0 & 0 \end{array}\right]$ and $\left[\begin{array}{ccc} \cos75^{{}^{\circ}} & \sin75^{{}^{\circ}} & 0 \end{array}\right]$ and with relative weights 2 : 1, (i) two fibers with orientations $\left[\begin{array}{ccc} 1 & 0 & 0 \end{array}\right]$ and $\left[\begin{array}{ccc} \cos60^{{}^{\circ}} & \sin60^{{}^{\circ}} & 0 \end{array}\right]$ and with relative weights 1 : 1, and (j) three perpendicular fibers with orientations $\left[\begin{array}{ccc} 1 & 0 & 0 \end{array}\right]$, $\left[\begin{array}{ccc} 0 & 1 & 0 \end{array}\right]$, and $\left[\begin{array}{ccc} 0 & 0 & 1 \end{array}\right]$. Tensors (a)–(j) correspond to **t**_0_^1^–**t**_0_^10^ in Table 4.

**Figure 2:**
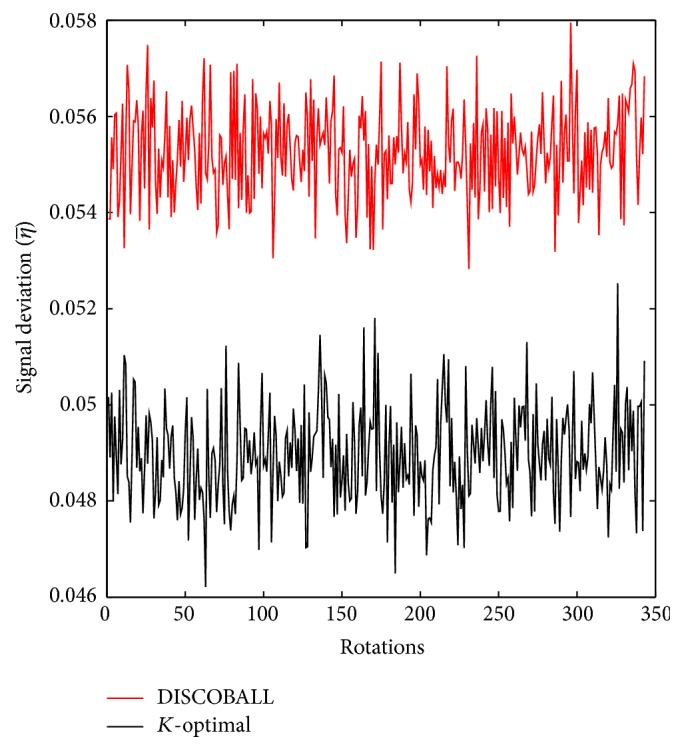
Results of rotational variance test for **t**_0_ = **t**_0_^1^ (*N* = 30): mean signal deviation $\bar{\eta }$ (vertical axis) is computed using Algorithm 1 given in the Appendix. The horizontal axis denotes 343 rotation matrices described in Section 4.2. Signal deviation of the *K*-optimal GES is consistently lower than that of the DISCOBALL scheme [26].

Signal deviation is defined in [16] to measure the rotational variance of a GES. As the diffusion tensor is reoriented, the accuracy and precision of the estimated parameters may vary. Knowledge of the rotational variance is thus very important in the dMRI community. For details see chapter 15 in [20]. Signal deviation is defined as [16]

(17) $$\begin{array}{c} \eta =\frac{1}{N}\overset{N}{\underset{i=1}{\sum }}\frac{\left|S\left(g_{i}\right)-\hat{S}\left(g_{i}\right)\right|}{S_{0}}, \end{array}$$

where *S*(**g**_*i*_) is the measured signal and $\hat{S}(g_{i})$ is the signal produced by the estimated tensor $\left(\hat{t}\right)$. To evaluate the rotational variance of a GES we select 343 rotation matrices. These matrices are obtained by taking equally spaced steps in each of *θ*, *ϕ*, and *ψ* and computing **R** = **R**_*x*_(*θ*)**R**_*y*_(*ϕ*)**R**_*z*_(*ψ*). To rotate fourth-order tensors, we use the approach in [16]. We evaluated the signal deviation using Algorithm 1 given in the Appendix, where we used the following setup for the Monte Carlo simulations: *N* = 30, *b* = 1500 s/(mm^2^), *N*_MC_ = 200 (number of Monte Carlo trials), SNR = *S*_0_/*σ* = 12.5, *N*_*R*_ = 343 (number of rotations), and **t**_0_ = **t**_0_^*i*^ × 10^−4^, *i* = 1,…, 10. All tensors used in the evaluation are listed in Table 4 and are plotted in Figure 1. The software in [28] is used to plot fourth-order tensors. As it can be seen in Figure 1, three tensors (a)–(c) correspond to single-fiber microstructures while six tensors (d)–(i) represent two crossing fibers (with different crossing angle and weights of the lobes) and the tensor in (j) shows three perpendicular fibers. Crossing angles below 60 degrees are not considered as it is known that fourth-order tensors cannot resolve such fiber architectures [29]. In Figure 2, the average signal deviation over Monte Carlo trials ($\bar{\eta }$) is plotted as a function of tensor orientation for the top two GESs (based on the condition number). It shows that, for **t**_0_^1^, signal deviation of the *K*-optimal GES is consistently less than that of the DISCOBALL scheme. The mean value and standard deviation of the $\bar{\eta }$ (over rotations) for all evaluated GESs/tensors are given in Table 5. It can be seen that, in all cases, *K*-optimal GES has the minimum mean value of signal deviation (corresponding numbers are denoted by bold font). Considering **t**_0_^1^, **t**_0_^8^, **t**_0_^9^, and **t**_0_^10^ the standard deviation of $\bar{\eta }$ is almost the same for all GESs. For **t**_0_^2^ to **t**_0_^7^, the *K*-optimal GES has the maximum $\sigma (\bar{\eta })$. However, even in these cases mean signal deviation of the *K*-optimal GES is far better than that of others. Thus, the proposed *K*-optimal GES is the most favorable choice in all cases.

**Figure 3:**
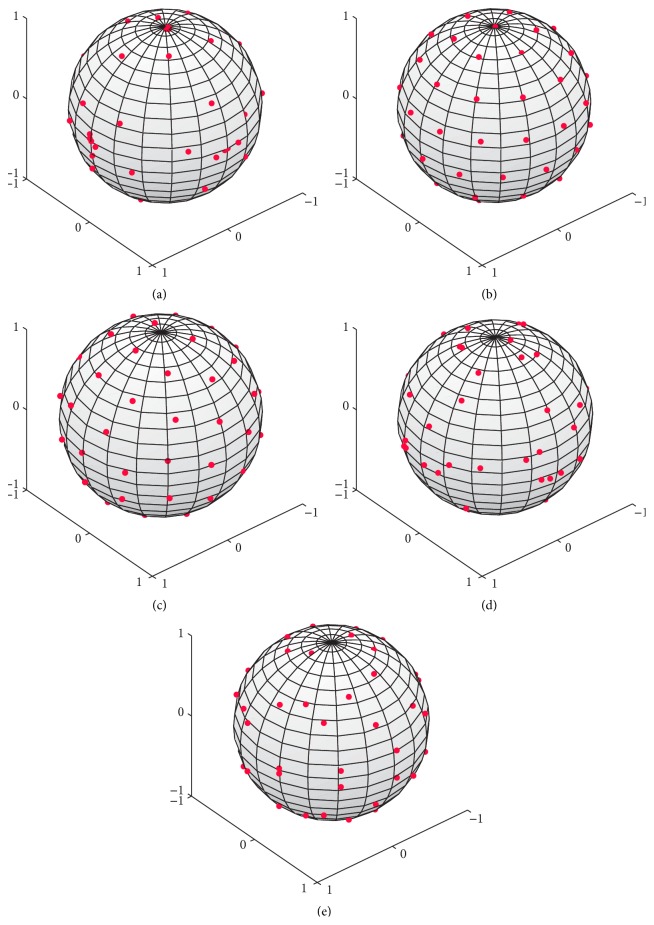
Distribution of directions over the unit sphere for different GESs (*N* = 30): (a) *K*-optimal [Proposed], (b) DISCOBALL [26], (c) Jones [20], (d) MCN [12], and (e) Wong and Roos [27].

The distribution of gradient encoding directions over the unit sphere for the evaluated GESs is plotted in Figure 3. The DISCOBALL and Jones schemes produce an approximately uniform (equidistance) distribution of points while the *K*-optimal, MCN, and Wong schemes produce a nonuniform distribution of points. Regarding the effect of uniform distribution of points, an interesting observation is that uniformly distributed GESs (the Jones and DISCOBALL) have minimum $\sigma (\bar{\eta })$ except for **t**_0_^2^ and **t**_0_^9^ (see Table 5). However, they do not lead to an overall better performance (as the *K*-optimal GES performs far better in terms of mean $\bar{\eta }$).

## 5. Discussions

The proposed approach can be applied in experiment design for other tensors, although the current work focuses on its application and results on fourth-order tensor estimation. In Section 2, the order of the diffusion tensor, *n*, can be any even natural number (to ensure antipodal symmetry). The extension to higher order tensors is possible using the same strategy (the dimension of the the information matrix in (13) will increase to (*n* + 1)(*n* + 2)/2).

In Section 2, we assumed that the noise (on *s*_*i*_) is zero mean and independent with constant variance. We acknowledge that, in the dMRI context, these noise assumptions may not hold, in general. However, our Monte Carlo simulations in Section 4 show that, for realistic cases (Rician noise on *S*(**g**_*i*_) and anisotropic tensors), the proposed GES yields the minimum signal deviation and the minimum rotational variance. Moreover, minimizing the condition number of the information matrix is motivated in [11, 12] regardless of the noise distribution. The condition number describes, without any assumptions about the noise distribution, how the noise in measurements propagates to the noise in diffusion tensor [11].

As we mentioned above the *K*-optimal design is not unique. This raises several questions including (i) why should we favor 60-point *K*-optimal design over four repetitions of 15-point *K*-optimal designs? and (ii) why should we favor a 60-point *K*-optimal design over a union of 40- and 20-point designs? To answer these types of questions further extensive studies and experiments with real data (or Monte Carlo simulations) are required (as in [30–32]) which is beyond the scope of this paper.

## 6. Conclusion

We showed that the *K*-optimal GES design for HOT estimation can be formulated as a nonconvex experiment design problem. Next, we solved the problem using convex relaxation and semidefinite programming. We also showed that resulting solutions have the following properties: (i) the proposed solution is the globally optimal solution; (ii) the obtained solutions are not unique, in general; (iii) nonzero entries of the optimal information matrix are proportional to the total number of measurements; (iv) odd moments of the *K*-optimal design must be zero; and (v) union of *K*-optimal solutions with *N*_1_ and *N*_2_ measurements leads to the *K*-optimal design for *N*_1_ + *N*_2_ measurements. Another advantage of this work is that it establishes a theoretical foundation for the experiment design in even order diffusion tensor imaging.

## Figures and Tables

**Algorithm 1:**
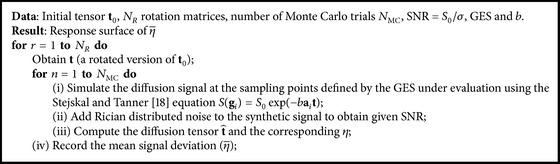
Pseudoalgorithm to compute response surface of $\overset{-}{\eta }$.

**Table 1:** Optimal gradient encoding scheme (**g**_*i*_s) for HOT estimation (*N* = 30).

*x*_*i*_	*y*_*i*_	*z*_*i*_	*x*_*i*_	*y*_*i*_	*z*_*i*_	*x*_*i*_	*y*_*i*_	*z*_*i*_
0.1514	−0.9883	−0.0161	0.3527	−0.8791	0.3207	−0.9125	0.2478	−0.3253
0.4840	0.1736	−0.8576	−0.0048	1.0000	−0.0068	−0.0163	0.0245	0.9996
−0.5357	0.0645	−0.8419	0.9960	−0.0292	0.0842	0.0160	0.0349	0.9993
−0.0633	−0.1941	0.9789	0.8959	−0.1044	−0.4317	0.3204	−0.3626	−0.8751
−0.4457	−0.8893	−0.1024	−0.0111	0.0185	0.9998	−0.1819	−0.8503	0.4938
−0.8564	−0.4798	0.1908	−0.1289	0.4227	−0.8970	0.0248	0.9996	−0.0146
0.9998	0.0123	0.0169	0.9988	−0.0129	0.0481	0.1318	0.9903	−0.0441
0.8391	−0.5377	−0.0829	0.0341	0.9994	−0.0089	−0.0149	−0.0427	−0.9990
−0.2315	−0.3334	−0.9139	0.0851	0.8468	0.5251	0.8780	0.3205	0.3556
0.3072	−0.9185	−0.2490	0.9867	0.0077	−0.1623	0.9973	0.0662	−0.0304

**Table 2:** *K*-optimal GES for second-order DTI using the proposed method (*N* = 6).

*x*_*i*_	*y*_*i*_	*z*_*i*_
0.9096	0.0000	0.4155
0.0000	0.4155	0.9096
0.4155	0.9096	0.0000
0.0000	0.4155	−0.9096
0.4155	−0.9096	0.0000
−0.9096	0.0000	0.4155

**Table 3:** Comparison of the proposed *K*-optimal GES with some existing methods in terms of condition number of the information matrix (*N* = 30).

GES	*K*-optimal	DISCOBALL	Jones	MCN	Wong
$\sqrt{\kappa (M)}$	1.9141	3.6392	3.8039	4.9473	4.9849
Reference	[Proposed]	[26]	[20]	[12]	[27]

**Table 4:** Ten-fourth-order tensors used for evaluation of the proposed method. These tensors correspond to different fiber architectures as illustrated in Figure 1.

**t**_0_^1^	**t**_0_^2^	**t**_0_^3^	**t**_0_^4^	**t**_0_^5^	**t**_0_^6^	**t**_0_^7^	**t**_0_^8^	**t**_0_^9^	**t**_0_^10^
2	0.60	0.54	0.73	0.64	0.45	0.69	0.56	0.70	8.50
0	0	0	0	0	0	0	0	0	0
1	0.38	0	0	0.03	0.79	0.29	0.84	0.34	0
0	0	0	0	0	0	0	0	0	0
2	13.31	23.77	12.51	8.66	5.47	10.86	7.48	7.25	8.50
0	0	0	0	0	0	0	0	0	0
0	−1.23	0	0	0	0	0.13	0.38	−0.05	0
0	0	0	0	0	0	0	0	0	0
0	30.02	0	0	0	0	10.37	6.80	15.23	0
3	0.99	2.16	0	0	0	0.02	0.04	0.01	0
0	0	0	0	0	0	0	0	0	0
3	27.03	0	0	0	0	3.93	2.32	12.49	0
0	0	0	0	0	0	0	0	0	0
0	9.70	0	0	0	0	0.69	0.35	4.35	0
17	1.83	0.29	12.27	16.20	19.33	12.32	16.18	13.01	8.50

**Table 5:** Comparison of the proposed *K*-optimal GES with some existing methods in terms of signal deviation (*N* = 30).

Tensor	*K*-optimal	DISCOBALL	Jones	MCN	Wong
Mean$\left(\overset{-}{\eta })[t_{0}^{1}\right]$	**0**.**0490**	0.0553	0.0552	0.0525	0.0527
$\sigma (\overset{-}{\eta })[t_{0}^{1}]$	0.0010	0.0009	0.0009	0.0012	0.0011
Mean$\left(\overset{-}{\eta })[t_{0}^{2}\right]$	**0**.**1410**	0.2083	0.2155	0.1524	0.1769
$\sigma (\overset{-}{\eta })[t_{0}^{2}]$	0.0457	0.0311	0.0310	0.0341	0.0281
Mean$\left(\overset{-}{\eta })[t_{0}^{3}\right]$	**0**.**0557**	0.0648	0.0648	0.0598	0.0607
$\sigma (\overset{-}{\eta })[t_{0}^{3}]$	0.0033	0.0015	0.0017	0.0029	0.0026
Mean$\left(\overset{-}{\eta })[t_{0}^{4}\right]$	**0**.**0481**	0.0541	0.0538	0.0512	0.0515
$\sigma (\overset{-}{\eta })[t_{0}^{4}]$	0.0016	0.0009	0.0009	0.0015	0.0010
Mean$\left(\overset{-}{\eta })[t_{0}^{5}\right]$	**0**.**0491**	0.0555	0.0553	0.0525	0.0527
$\sigma (\overset{-}{\eta })[t_{0}^{5}]$	0.0016	0.0009	0.0010	0.0017	0.0012
Mean$\left(\overset{-}{\eta })[t_{0}^{6}\right]$	**0**.**0507**	0.0579	0.0577	0.0544	0.0546
$\sigma (\overset{-}{\eta })[t_{0}^{6}]$	0.0018	0.0011	0.0011	0.0020	0.0015
Mean$\left(\overset{-}{\eta })[t_{0}^{7}\right]$	**0**.**0599**	0.0723	0.0725	0.0651	0.0663
$\sigma (\overset{-}{\eta })[t_{0}^{7}]$	0.0032	0.0020	0.0022	0.0031	0.0029
Mean$\left(\overset{-}{\eta })[t_{0}^{8}\right]$	**0**.**0546**	0.0642	0.0639	0.0594	0.0597
$\sigma (\overset{-}{\eta })[t_{0}^{8}]$	0.0016	0.0011	0.0012	0.0021	0.0015
Mean$\left(\overset{-}{\eta })[t_{0}^{9}\right]$	**0**.**0796**	0.1096	0.1081	0.0879	0.0930
$\sigma (\overset{-}{\eta })[t_{0}^{9}]$	0.0122	0.0102	0.0091	0.0109	0.0083
Mean$\left(\overset{-}{\eta })[t_{0}^{10}\right]$	**0**.**0440**	0.0476	0.0474	0.0461	0.0466
$\sigma (\overset{-}{\eta })[t_{0}^{10}]$	0.0008	0.0007	0.0007	0.0008	0.0007

## Appendix

### Pseudocode for the Algorithm Used in Section 4.2

See Algorithm 1.
